# Four new species of *Trichoderma* in the *Harzianum* clade from northern China

**DOI:** 10.3897/mycokeys.73.51424

**Published:** 2020-10-08

**Authors:** Xin Gu, Rui Wang, Quan Sun, Bing Wu, Jing-Zu Sun

**Affiliations:** 1 School of Agriculture, Ningxia University, Yinchuan, Ningxia 750021, China Ningxia University Yinchuan China; 2 State Key Laboratory of Mycology, Institute of Microbiology, Chinese Academy of Sciences, No. 3 Park 1, Beichen West Road, Chaoyang District, Beijing 100101, China Chinese Academy of Sciences Beijing China

**Keywords:** compost, fungicolous, Hypocreaceae, mycoparasite

## Abstract

The *Harzianum* clade of *Trichoderma* comprises many species, which are associated with a wide variety of substrates. In this study, four new species of *Trichoderma*, namely *T. lentinulae*, *T. vermifimicola*, *T. xixiacum*, and *T. zelobreve*, were encountered from a fruiting body and compost of *Lentinula*, soil, and vermicompost. Their colony and mycelial morphology, including features of asexual states, were described. For each species, their DNA sequences were obtained from three loci, the internal transcribed spacer (ITS) regions of the ribosomal DNA, the gene encoding the second largest nuclear RNA polymerase subunit (RPB2), the translation elongation factor 1-α encoding gene (TEF1-α). The analysis combining sequences of the three gene regions distinguished four new species in the *Harzianum* clade of *Trichoderma*. Among them, *T. lentinulae* and *T. xixiacum* clustered with *T. lixii*, from which these new species differ in having shorter phialides and smaller conidia. Additionally, *T. lentinulae* differs from *T. xixiacum* in forming phialides with inequilateral to a strongly-curved apex, cultural characteristics, and slow growth on PDA. *Trichoderma vermifimicola* is closely related to *T. simmonsii*, but it differs from the latter by producing phialides in verticillate whorls and smaller conidia. *Trichoderma zelobreve* is the sister species of *T. breve* but is distinguished from *T. breve* by producing shorter and narrower phialides, smaller conidia, and by forming concentric zones on agar plates. This study updates our knowledge of species diversity of *Trichoderma*.

## Introduction

The genus *Trichoderma* Pers., introduced by Persoon (1794), is cosmopolitan, including saprotrophs and mycoparasites in a diversity of ecosystems, such as agricultural fields, prairies, forests, salt marshes, and fungal fruiting body (Gazis and Chaverri 2010; Chaverri et al. 2015; Qiao et al. 2018). Species of this genus have been widely used in the biocontrol of plant pathogens (Chaverri et al. 2015; Degenkolb et al. 2015; Bunbury-Blanchette and Walke 2019) and production of enzymes and bioactive compounds (Sun et al. 2016). Nevertheless, some of them are associated with green mold diseases in the commercial production of mushrooms (Innocenti et al. 2019; Sun et al. 2019a) . Morphologically, the asexual-morphs are similar in producing branched tree-like conidiophores with cylindrical to nearly subglobose phialides and ellipsoidal to globose conidia, but their variation is insufficient to differentiate the *Trichoderma* species (Chaverri et al. 2015; Qin and Zhuang 2017; Qiao et al. 2018). Multilocus molecular phylogeny, based on combined sequence data of the internal transcribed spacer (ITS) regions, RNA polymerase II subunit (RPB2), and the translation elongation factor 1-α gene (TEF1-α), enables rapid and accurate identification of the *Trichoderma* species (Druzhinina et al. 2005; Atanasova et al. 2013; Chaverri et al. 2015). Currently, the combination of multi-gene phylogenetic analysis and phenotypic characteristics is extensively applied in species delimitation of *Trichoderma* (du Plessis et al. 2018; Qiao et al. 2018; Innocenti et al. 2019).

*Trichoderma harzianum* Rifai is one of the most well-known *Trichoderma* species, due to its antifungal properties and effective bio-control ability, used to suppress soil-borne plant pathogens (Chaverri et al. 2015; Degenkolb et al. 2015; Bunbury-Blanchette and Walker 2019). As a cosmopolitan and ubiquitous fungus, it has been isolated from diverse substrates, such as soil, plant tissue, and mushrooms (Chaverri et al. 2015; Jaklitsch and Voglmayr 2015; Innocenti et al. 2019; Sun et al. 2019b). Since Chaverri et al. (2015) provided a systematic revision of species in the *Harzianum* clade, numerous new species have been described (Jaklitsch and Voglmayr 2015; Qin and Zhuang 2016a; Sun et al. 2016; Chen and Zhuang 2017b; Qiao et al. 2018). Currently, more than 60 species are placed in the *Harzianum* clade (Jaklitsch and Voglmayr 2015; Qin and Zhuang 2016a, b, 2017; Chen and Zhuang 2017b; Qiao et al. 2018; Phookamsak et al. 2019;) .

It is estimated that 136 new species of *Trichoderma* have been recognised since 2015 (www.indexfungorum.org 2020), with 84 among these reported from China (Sun et al. 2012; Qin and Zhuang 2016a, b, 2017; Chen and Zhuang 2017a, b; Qiao et al. 2018), which evidenced that China has a high species diversity of *Trichoderma* (Zhu and Zhuang 2015; Jiang et al. 2016). In our survey of *Trichoderma*, eighteen isolates were obtained from soil, mushroom substrates, and vermicompost from northern China. Four new species belonging to the *Harzianum* clade were identified based on morphological features and DNA sequence data at three loci: the genes encoding RNA polymerase II subunit (RBP2) and translation elongation factor 1-α gene (TEF1-α), and the internal transcribed spacer (ITS) regions of the nuclear ribosomal RNA gene.

## Materials and methods

### Sampling sites and strains isolation

Since *Trichoderma* is easily isolated from soil, mushroom substrates, and earthworm substrates, the soil, mushroom substrates, and earthworm were therefore collected from Yinchuan, Ningxia Hui Autonomous Region, and Chaoyang district, Beijing, China. All the samples were stored at 4 °C before fungal isolation. *Trichoderma* strains were isolated by gradient dilution and the spread plate method or directly from the mushroom substrates. Three dilutions (10^-1^, 10^-2^, and 10^-3^) were prepared with 1 g soil and sterile water, and 100 µl of each dilution was spread on a 9 cm diameter Petri dish of PDA agar with100 mg/L chloramphenicol added. The plates were then incubated at 25 °C. Each of the individual colonies was transferred to a new PDA dish after 1–3 days and incubated at 25 °C. Dried cultures from the single spore or specimens of new species were deposited in the Herbarium Mycologicum Academiae Sinicae (HMAS) and the ex-type strains were preserved in the China General Microbiological Culture Collection Center (CGMCC)

### Morphological analysis

For morphological studies, we used three different media: cornmeal dextrose agar (CMD, Difco, BD Science, USA), PDA (Difco, BD Science, USA), and synthetic low nutrient agar (SNA, Difco, BD Science, USA) (Chaverri et al. 2015). Each strain was first cultured on an SNA plate for 3 days and a small agar piece of 0.5 cm diameter with mycelium was then transferred, respectively, to new CMD, PDA, and SNA plates. Strains were incubated in 9 cm diam with three replicates. Petri dishes at 25 °C with a 12 h natural light and 12 h darkness interval. Colony diameter at 25 °C was measured three days after inoculation, and the time when mycelium entirely covered the surface of the agar plate was also recorded. Micromorphological characters were examined from the cultures of one-week-old colonies on SNA (Chaverri et al. 2015). A Nikon Ellipse 80i light microscope, equipped with differential interference contrast (DIC) optics, was used to capture digital images.

### DNA extraction, PCR and sequencing

Genomic DNA of each strain was extracted from fresh mycelium growing on PDA after 5 days of growth following the rapid “thermolysis” method described in Zhang et al. (2010). For the amplification of ITS, RPB2, and TEF1-α gene fragments, ITS4 and ITS5 for ITS (White et al. 1990), EF1-728F (Carbone and Kohn 1999) and TEF1LLErev (Jaklitsch et al. 2005) for TEF1, and RPB2-5F and RPB2-7R for rpb2 (Liu et al. 1999) were used. Each PCR reaction consisted of 12.5 μl T5 Super PCR Mix (containing Taq polymerase, dNTP, and Mg^2+^, Beijing TsingKe Biotech Co. Ltd., Beijing), 1.0 μl of forward primer (10 μM), 1.0 μl of reverse primer (10 μM), 0.5 μl DMSO, 3 μl DNA template and 7 μl double sterilized water. PCR reactions were in Eppendorf Mastercycler, following the protocols described by Sun et al. (2016). PCR products were purified with the PCR product purification kit (TIANGEN Biotech, Beijing, China), and sequencing was carried out in both directions on an ABI 3730 XL DNA sequencer (Applied Biosystems, Foster City, California) with primers used during PCR amplification.

### Phylogenetic analyses

Preliminary BLAST searches with ITS, RPB2, and TEF1-α gene sequences of the new isolates against NCBI, TrichOKey (Druzhinina and Kopchinski 2006), and TrichoBlast (Kopchinskiy et al. 2005) databases identified species closely related to our isolates. Based on this information, sequences of ITS, RBP2, and TEF1-α of 133 strains, representing 59 species were downloaded from GenBank, following recent publications (Qin and Zhuang 2017; Qiao et al. 2018; Innocenti et al. 2019). Among them, 139 strains are belonging to the *Harzianum* clade, and *Trichoderma ceramicum*, *T. parestonicum*, and *T. estonicum* were chosen to represent the outgroup.

Tree alignment files were generated by using MAFFT version 7.03 with the Q-INS-I strategy (Katoh and Standley 2013). Conserved blocks were selected from the initial alignments with Gblocks 0.91 b (Castresana 2000). The appropriate nucleotide substitution model for each gene was determined by using MrModeltest v2.4 (Nylander 2004). HKY + I + G was estimated as the best-fit model for RPB2, and GTR + I + G was estimated as the best-fit model for TEF1-α and ITS under the output strategy of AIC. The partition homogeneity test (*p* = 0.01) indicated that the individual partitions were not significantly incongruent (Cunningham 1997), thus the aligned sequences of ITS, RPB2, and TEF1-α were combined for analyses. The multi-locus phylogenetic analyses included 1065 characters for RBP2, 587 characters for TEF1-α, and 555 characters for ITS. All characters were weighted equally and gaps were treated as missing characters.

Maximum Likelihood (ML) analyses were performed by RAxML (Stamatakis 2006), using the GTR-GAMMA-I model. The maximum likelihood bootstrap proportions (MLBP) were using 1000 replicates. Bayesian Inference (BI) analyses were conducted with MrBayes v3.2.6 (Ronquist et al. 2012). Metropolis-coupled Markov Chain Monte Carlo (MCMC) searches were calculated for 10,000,000 generations, sampling every 100^th^ generation with the best best-fit model for each gene. Two independent analyses with six chains each (one cold and five heated) were carried out until the average standard deviation of the split frequencies dropped below 0.01. The initial 25% of the generations of MCMC sampling were discarded as burn-in. The refinement of the phylogenetic tree was used for estimating Bayesian inference posterior probability (PP) values. The Tree was viewed in FigTree v1.4 (Rambaut 2012), values of Maximum likelihood bootstrap proportions (MLBP) greater than 50% and Bayesian inference posterior probabilities (BIPP), greater than 95% at the nodes, are shown along branches. The final alignments and the trees obtained have been deposited in TreeBASE (TreeBASE accession number: 25400).

## Results

### Phylogeny

The preliminary BLAST searches with ITS, RPB2, and TEF1-α gene sequences of the new isolates suggest our isolates were highly similar to species from *Trichoderma* in the *Harzianum*-complex. Therefore, as the next step phylogenetic analyses were conducted by using a single gene of ITS, RPB2, TEF1-α, and multi-gene dataset of cascaded ITS, RPB2, and TEF1-α, respectively. The phylogenetic trees showed that our isolates were placed in the *Harzianum* clade (Fig. 1, Suppl. material 1: Fig. S1, Suppl. material 2: Fig. S2, Suppl. material 3: Fig. S3). In the phylogenetic tree conducted by a combined matrix of ITS, RPB2, and TEF1-α sequences, isolates of *T. lentinulae*, *T. xixiacum*, and *T. lixii* formed a well-supported clade (MLBP/BIBP = 73%/1.00). Within this clade, isolates of *T. lentinulae* and *T. xixiacum* formed a subclade with maximum support. Isolates of *T. vermifimicola* clustered together with *T. simmonsii* (BIBP = 1.00), both forming a subclade with maximum support (MLBP/BIBP = 100%/1.00, Fig. 1). *Trichoderma zelobreve* and *T. breve*, were distinguished by maximum support to respective clades while forming a highly supported clade (MLBP/BIBP = 100%/1.00, Fig. 1).

**Figure 1.**
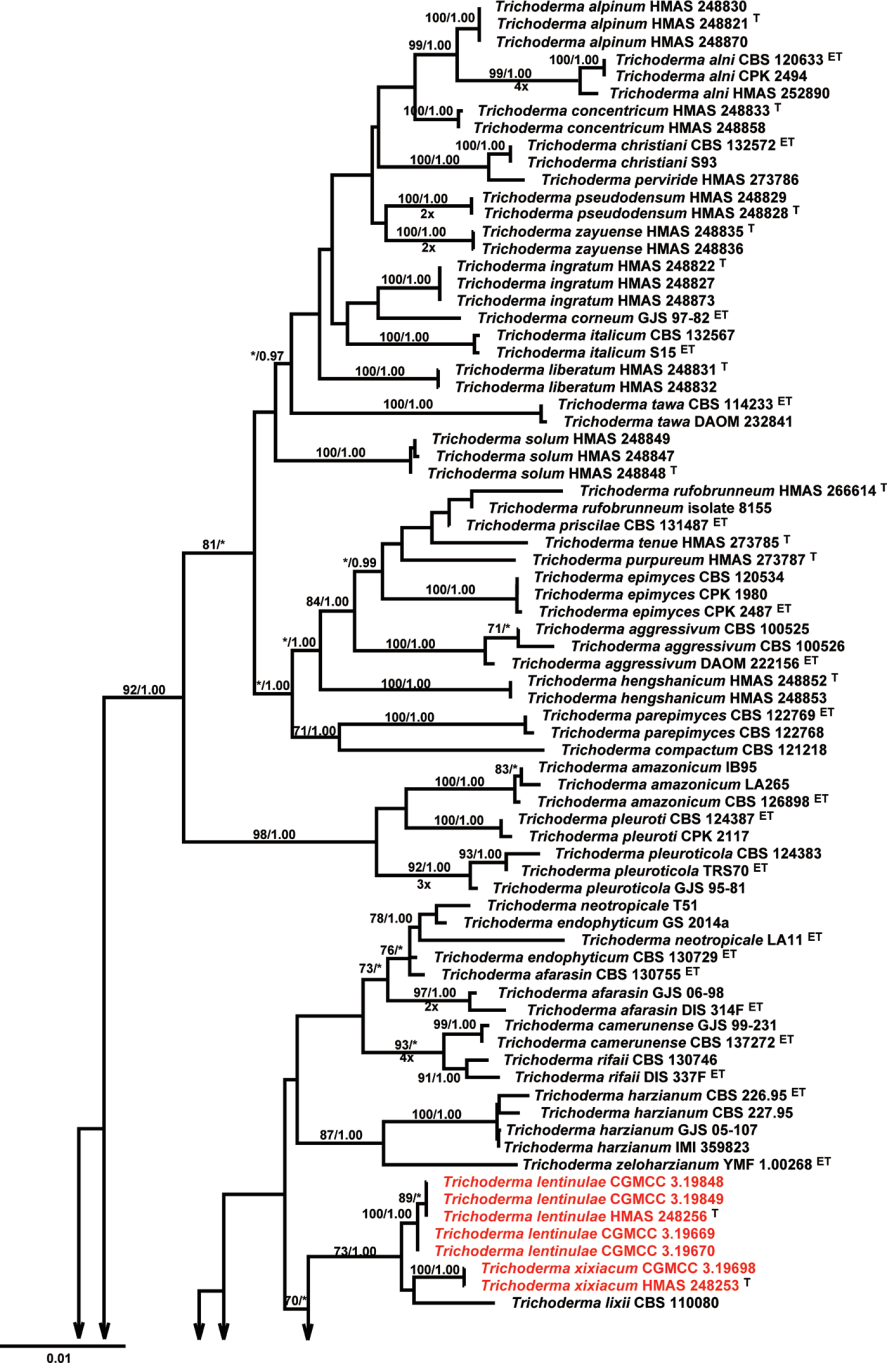
Phylogenetic tree based on Maximum Likelihood analysis of a combined ITS, RPB2, and TEF1α sequence dataset. *Trichoderma estonicum*, *Trichoderm parastinicum*, *Trichoderm ceramicum* were chosen as the outgroup. Bootstrap Values higher than 70% from RAxML (BSML) (left) and Bayesian posterior probabilities greater than 0.95 (BYPP) (right) are given above the nodes. ^T^ indicates the type; ^ET^ indicates the ex-living type. Isolates obtained in this study are in red.

**Figure 1.**
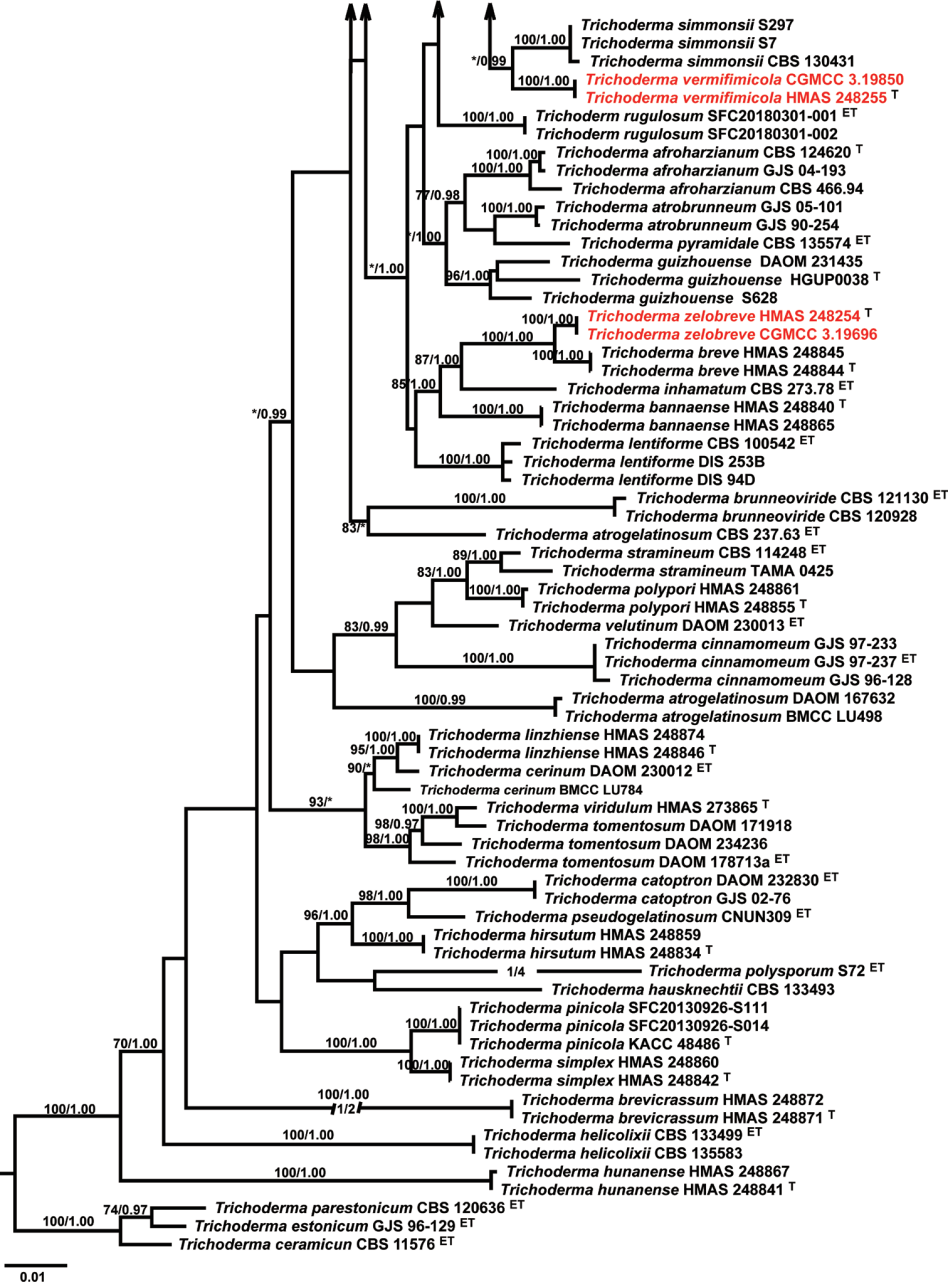
Continued.

The ITS gene could not distinguish our isolates from other species within the *Harzianum* clade (Suppl. material 1: Fig. S1). In the phylogenetic tree resulted from the RPB2 gene, *Trichoderma lentinulae*, *T. xixiacum*, and *T. lixii* formed a highly supported clade (MLBP/BIBP = 100%/1.00), but within this clade, *T. lentinulae*, *T. xixiacum* were not distinguished (Suppl. material 2: Fig. S2). Isolates of *T. vermifimicola* formed a distinct clade (MLBP/BIBP = 100%/1.00) and grouped with *T. simmonsii*, *T. guizhouense*, and *T. rugulosum* but weakly supported (Suppl. material 3: Fig. S3). *Trichoderma zelobreve* and *T. breve* also formed a highly supported clade (MLBP/BIBP = 98%/1.00), but *T. zelobreve* and *T. breve*, were distinguished by maximum support to respective clades while forming a highly supported clade (MLBP/BIBP = 100%/1.00, Suppl. material 2: Fig. S2). In the phylogenetic tree resulted from the TEF1-α gene, *T. zelobreve* and *T. breve* also formed a highly supported clade (MLBP/BIBP = 98%/1.00), but were not distinct from each other (Suppl. material 3: Fig. S3). Isolates of *T. lentinulae*, *T. xixiacum*, *T. vermifimicola*, and *T. simmonsii* clustered together but this clade was not well-supported. Within this clade, isolates of *T. lentinulae* formed a well-supported subclade (MLBP/BIBP = 91%/1.00). *Trichoderma xixiacum* and *T. vermifimicola* formed a highly supported subclade (MLBP/BIBP = 100%/1.00). Within this group, isolates of *T. vermifimicola* clustered together with well-supported (MLBP/BIBP = 93%/1.00, Suppl. material 3: Fig. S3).

## Taxonomy

### Trichoderma lentinulae

Taxon classificationFungiHypocrealesHypocreaceae

Jing Z. Sun & X.Z. Liu sp. nov.

0AA38324-0ACC-5EC9-87D7-CD71AAE0401C

833233

Fig. 2

#### Etymology.

Latin, *lentinulae*, refers to the host from which the fungus was isolated.

#### Type.

China. Haidian District, Beijing, 39°57'40"N, 116°19'40"E, ca. 27 m elev., from a fruiting body and mushroom spawn of *Lentinula edodes*, 19 Oct 2018, Jing Z. Sun (HMAS 248256, holotype), ex-type culture CGMCC 3.19847.

#### Description.

**Figure 2.**
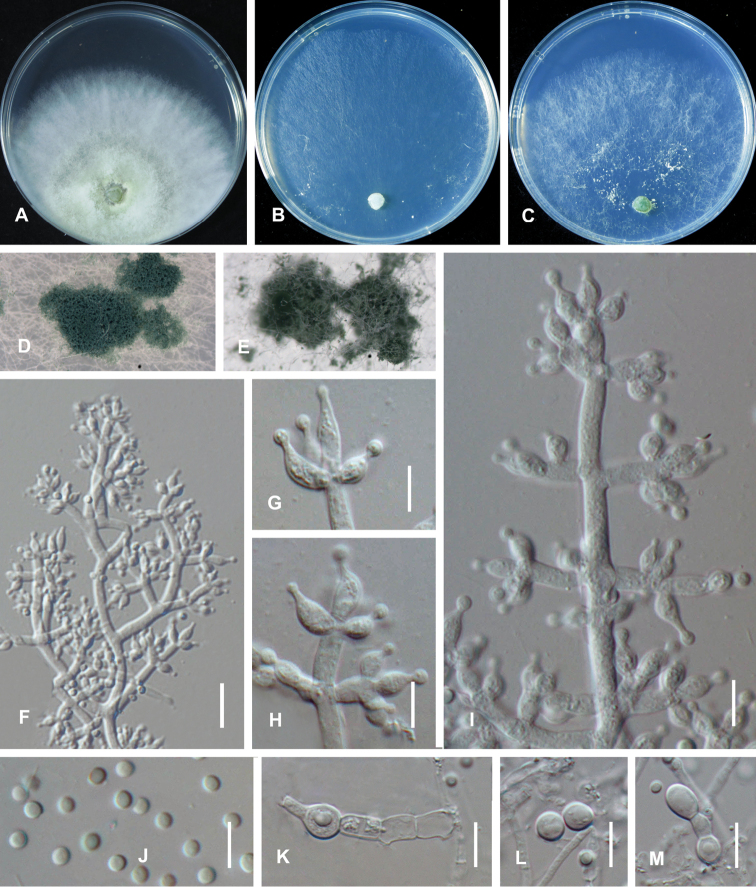
*Trichoderma lentinulae* (CGMCC 3.19847). Cultures at 25 °C after 3 days (**A** on PDA **B** on CMD**C** on SNA) **D** conidiation pustules on CMD after 10 days **E** conidiation pustules on CMD after 10 d **F** conidiophores **G–I** Conidiophores and phialides **J** conidia **K–M** chlamydospores. Scale bars: 25 µm (**F**); 10 µm (**G–M**).

On CMD after 72 h, colony radius 57–58 mm at 25 °C, covering the plate at 30 °C, 4–5 mm at 35 °C. Colony hyaline, weak, indistinctly radial. Aerial hyphae short, inconspicuous. No diffusing pigment noted, odor indistinct (Fig. 2B). Conidial production noted after 3 days, scant, effuse in aerial hyphae, becoming blue-green after 7 days. Chlamydospores not observed.

On PDA after 72 h, colony radius 45–46 mm at 25 °C, mycelium covering the plate at 30 °C, 11–12 mm at 35 °C. Colony white to yellowish-white, regularly circular, indistinctly zonate; mycelium dense and radial. No diffusing pigment, not distinct odor (Fig. 2A). Conidial production noted after 3 days, starting around the original inoculum, effuse in the aerial hyphae, first white, turning green after 3 d. Chlamydospores unobserved.

On SNA after 72 h, colony radius 51–52 mm at 25 °C, 52–53 mm at 30 °C, 4–5 mm at 35 °C. Colony hyaline, indistinctly zonate; mycelium loose, especially at the margin. Aerial hyphae loose. No diffusing pigment, not distinct odor (Fig. 2C). Conidial production noted after 2 days, starting around the inoculum, effuse in the aerial hyphae. Small pustules formed around the inoculum, first white, turning green after 3 d, with hairs protruding beyond the surface. Conidiophores pyramidal with opposing branches, less frequently solitary, closely-spaced branches, each branch, and the main axis terminating in 2–5 cruciately to nearly verticillately disposed phialides (Fig. 2F, H, I). Phialides ampulliform, typically strongly constricted below the tip, less frequently lageniform and then usually apex and inequilateral to strongly curved, hyaline, (3.5–)4.0–6.0(–6.5) × (2.0–)2.5–3.0(–3.5) µm (*x̄*= 4.5 × 3.0 μm, n = 30), length/width ratio (1.5–)2.0–3.0(–5.0) (*x̄*= 2.0, n = 30), base 1.0–2.5 μm (*x̄*= 1.5 μm)(Fig. 2G, H, I). Conidia ovoid to globose, smooth, hyaline when young, becoming green to dark green with age, (2.0–)2.5–3.0(–3.5) × (1.5–)2.0–2.5(–3.0) µm (*x̄*= 2.5 × 2.2 μm, n = 50), length/width ratio (1.0–)1.1–1.4 (–1.5) (*x̄*= 1.2, n = 50) (Fig. 2J). Chlamydospores common, apex or intercalary, ellipsoid or subglobose, (3.5–)5.0–6.5(–7.0) × (3.0–)4.0–5.0(–6.0) µm (*x̄*= 5.5 × 4.5 μm, n = 30), length/width ratio (1.0–)1.2–1.5 (–1.7) (*x̄*= 1.2, n = 30) (Fig. 2K–M).

#### Additional specimen examined.

China. Haidian District, Beijing, 39°57'40"N, 116°19'40"E, ca. 27 m elev., From a fruiting body and mushroom spawn of *Lentinula edodes*, 19 Oct 2018, Jing Z. Sun, living culture CGMCC 3.19848; Xixia District, Yinchuan, Ningxia Hui Autonomous Region, 38°38'52"N, 106°9'33"E, ca. 1127 m elev., from rhizosphere soil of Lycium chinois, 17 Oct 2018, Jing Z. Sun, living culture CGMCC 3.19699; ibid., living culture CGMCC 3.19670.

#### Teleomorph.

Undetermined.

#### Note.

The species is characterized by tree-like conidiophores, phialides verticillate or in whorls of 3–4, spindle-like to fusiform phialides (4.0–6.0 × 2.5–3.0 μm) and ovoid to subglobose conidia. Differs from *T. lixii* by shorter and wider phialides and smaller conidia. Differs from *Trichoderma xixiacum* by compact, relatively smaller phialides, and the pustules not forming distinctly zonate of pustules on SNA.

### Trichoderma vermifimicola

Taxon classificationFungiHypocrealesHypocreaceae

Jing Z. Sun & X.Z. Liu sp. nov.

F20EA222-A753-5CD4-ABBF-A000DCDE66A2

833234

Fig. 3

#### Etymology.

Latin, *vermifimicola*, refers to the habitat of the type species.

#### Type.

China. Yongning, Yinchuan, the Ningxia Hui Autonomous Region, 40°0'41"N, 116°23'37"E, ca. 1678 m elev., from the substrates for earthworm cultivation, 18 Oct 2018, Jing Z. Sun (HMAS 248255, holotype), ex-type culture CGMCC 3.19694.

#### Description.

**Figure 3.**
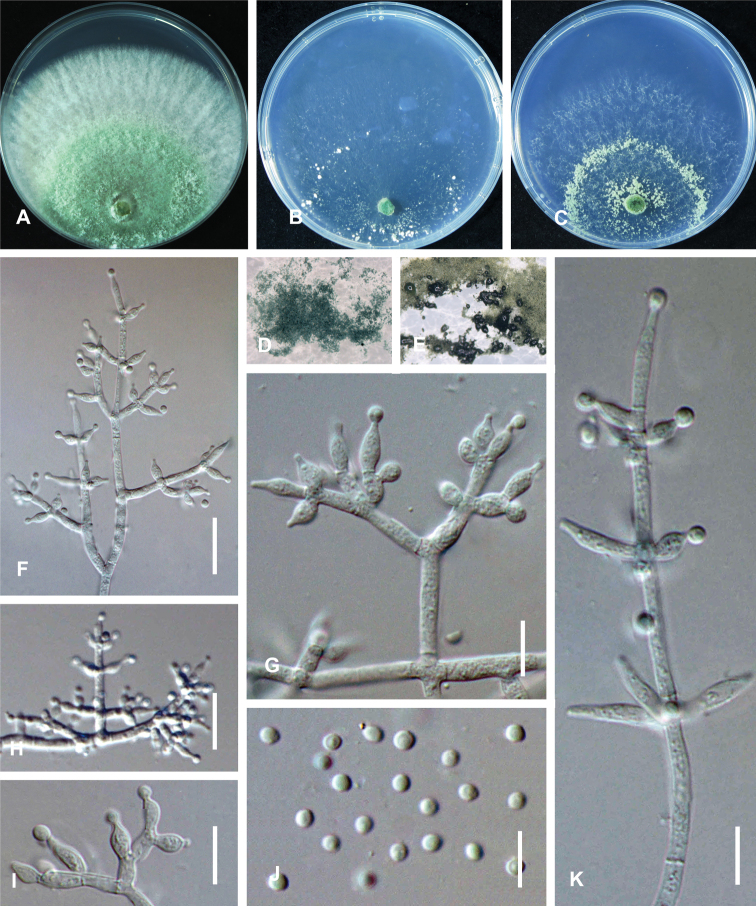
*Trichoderma vermifimicola* (CGMCC 3.19694). Cultures at 25 °C after 3 days (**A** on PDA **B** on CMD**C** on SNA) **D** conidiation pustules on CMD after 10 days **E** conidiation pustules on SNA after 10 d **F, H** conidiophores **G, J, K** conidiophores and phialides **I** conidia. Scale bars: 25 µm (**F, H**); 10 µm (**G, J–K**).

On CMD after 72 h, colony radius 49–51 mm at 25 °C, 51–52 mm at 30 °C, 4–5 mm at 35 °C. Colony hyaline, irregularly circular, indistinctly zonate; mycelium loose. Aerial hyphae short, inconspicuous. No diffusing pigment, not distinct odor. Conidial production noted after 3 days, starting around the inoculum (Fig. 3B). Small pustules formed at the colony margin, first white, turning blue-green after 7 d, with hairs protruding beyond the surface. Chlamydospores unobserved.

On PDA after 72 h, colony radius 55–58 mm at 25 °C, 55–56 mm at 30 °C, 5–6 mm at 35 °C. Colony white-green to bright green, regularly circular, distinctly zonate; mycelium dense and radial. Aerial hyphae short, inconspicuous. No diffusing pigment, not distinct odor. Conidial production noted after 2 days, starting around the inoculum, effuse in the aerial hyphae, first white, turning green after 2 d (Fig. 3A). Chlamydospores unobserved.

On SNA after 72﻿﻿ h, colony radius 48–50 mm at 25 °C, 51–52 mm at 30 °C, 3–4 mm at 35 °C. Colony hyaline, regularly circular, distinctly zonate; mycelium loose, especially at the margin. Aerial hyphae short, inconspicuous. No diffusing pigment, not distinct odor. Conidial production noted after 2 days, starting around the inoculum, effuse in the aerial hyphae. Small pustules formed along with two concentric rings, first white, turning yellow-green after 3 d, with hairs protruding beyond the surface (Fig. 3C). Conidiophores pyramidal with opposing branches, the distance between branches relatively large, each branch terminating in a whorl of 2–3 phialides, phialides sometimes solitary on the main axis (Fig. 3F, H, K); whorls typically cruciate, but often nearly verticillate (Fig. 3K); rarely conidiophores nodose and phialides disposed in more or less botryose clusters (Fig. 3H). Phialides ampulliform to lageniform, often constricted below the tip to form a narrow neck, hyaline, (4.4–)5.0–10.5(–11.2) × (2.0–)2.5–3.0(–3.5) µm (*x̄*= 6.6 × 2.7 μm, n = 30), length/width ratio (1.5–)1.8–2.8(–5.3) (*x̄*= 2.4, n = 30), base 1.6–2.5 μm (*x̄*= 1.9 μm) (Fig. 3G, I, K). Conidia ovoid to subglobose, smooth, hyaline when young, becoming green to dark green with age, (2.0–)2.3–2.6(–3.0) × (1.5–)2.0–2.4(–2.8) µm (*x̄*= 2.4 × 2.2 μm, n = 50), length/width ratio (1.0–)1.1–1.4(–1.7) (*x̄*= 1.2, n = 50) (Fig. 3J). Chlamydospores unobserved. No odor; no diffusing pigment observed.

#### Additional specimen examined.

China. Xixia District, Yinchuan, Ningxia Hui Autonomous Region, 38°38'52"N, 106°9'33"E, ca. 1127 m elev., from rhizosphere soil of Lycium chinois, 17 Oct 2018, Jing Z. Sun, living CGMCC 3.19697.

#### Teleomorph.

Undetermined.

#### Note.

Characterized by tree-like conidiophores, verticillate or in whorls of 3–4, ampulliform to lageniform phialides (5.0–10.5 × 2.5–3.0 μm), ovoid to subglobose conidia (2.4–2.6 × 2.0–2.5 μm). Differs from *Trichoderma simmonsii* by forming loose branches in whorls, relatively longer and thinner phialides, smaller conidia, and the fewer pustules on SNA.

### Trichoderma xixiacum

Taxon classificationFungiHypocrealesHypocreaceae

Jing Z. Sun & X.Z. Liu sp. nov.

E6B880C9-D090-5DE6-A633-F72AFF924701

833235

Fig. 4

#### Etymology.

Latin, *xixiacum*, refers to the type locality.

#### Type.

China. Xixia District, Yinchuan, Ningxia Hui Autonomous Region, 38°38'52"N, 106°9'33"E, ca. 1127 m elev., from rhizosphere soil of *Lycium chinois*, 17 Oct 2018, Jing Z. Sun (HMAS 248253, holotype), ex-type culture CGMCC 3.19697.

#### Description.

**Figure 4.**
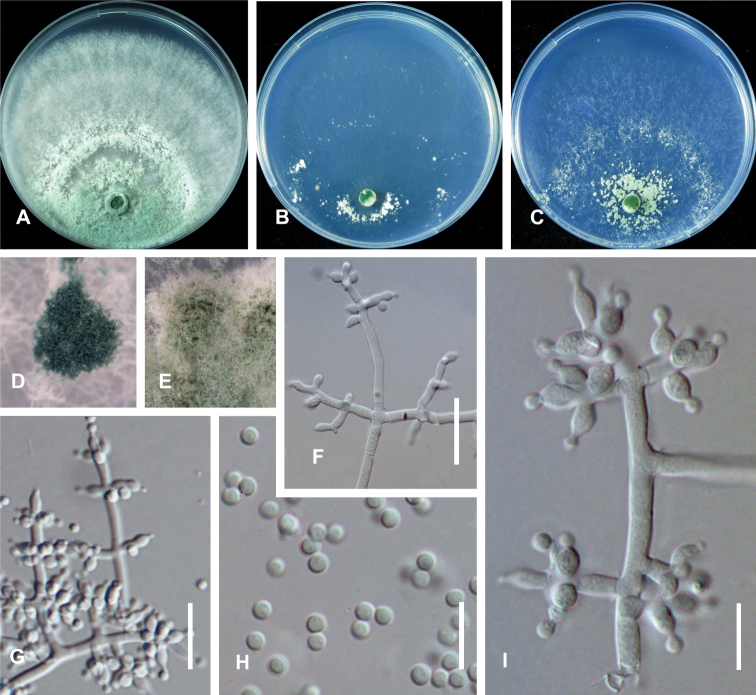
*Trichoderma xixiacum* (CGMCC 3.19697). Cultures at 25 °C after 3 d (**A** on PDA **B** on CMD**C** on SNA) **D** conidiation pustules on CMD after 10 d **E** conidiation pustules on SNA after 10 d **F, G, I** conidiophores and phialides **H** conidia. Scale bars: 10 µm (**F, G**); 10 µm (**H, I**).

On CMD after 72 h, colony radius 55–56 mm at 25 °C, covering the plate at 30 °C, 9–11 mm at 35 °C. Colony hyaline, indistinctly zonate, mycelia loose. Aerial hyphae short, inconspicuous. No diffusing pigment, not distinct odor (Fig. 4B). Conidial production noted after 3 days, effuse in aerial hyphae, becoming blue-green after 4 days. Chlamydospores unobserved.

On PDA after 72 h, colony radius 59–60 mm at 25 °C, covering the plate at 30 °C, 7–8 mm at 35 °C. Colony white to yellow-white, regularly circular, indistinctly zonate; mycelium dense and radial. Aerial hyphae conspicuous. No diffusing pigment, not distinct odor (Fig. 4A). Conidial production noted after 3 days, starting around the original inoculum, effuse in the aerial hyphae, first white, turning blue-green after 7 d. Chlamydospores unobserved.

On SNA after 72 h, colony radius 51–52 mm at 25 °C, 52–53 mm at 30 °C, 4–5 mm at 35 °C. Colony hyaline, indistinctly zonate; mycelium loose, especially at the margin. Aerial hyphae short. No diffusing pigment, not distinct odor (Fig. 4C). Conidial production noted after 2 days, starting around the inoculum, effuse in the aerial hyphae. Small pustules formed around the inoculum, first white, turning green after 3 d, with hairs protruding beyond the surface. Conidiophores pyramidal with opposing branches, less frequently solitary, closely-spaced branches, each branch, and the main axis terminating in 2–5 cruciately to nearly verticillately disposed phialides (Fig. 4F, G, I). Phialides ampulliform to lageniform, often constricted below the tip to form a narrow neck, hyaline, (3.2–)3.5–7.0(–9.3) × (2.3–)2.6–3.3(–3.6) µm (*x̄*= 5.0 × 3.0 μm, n = 50), length/width ratio (1.2–)1.5–2.5(–4) (*x̄*= 1.8, n = 50), base 1.6–2.2 μm (*x̄*= 1.8 μm, n = 50) (Fig. 4I). Conidia subglobose to globose, smooth, hyaline when young, becoming green to dark green with age, (2.0–)2.3–2.7(–3.0) × (1.6–)2.0–2.6(–3.0) µm (*x̄*= 2.5 × 2.2 μm, n = 50), length/width ratio 1.0–1.3(–1.7) (*x̄*= 1.1, n = 50) (Fig. 4H). Chlamydospores unobserved. No odor; no diffusing pigment observed.

#### Additional specimen examined.

China. Xixia District, Yinchuan, Ningxia Hui Autonomous Region, 38°38'52"N, 106°9'33"E, ca. 1127 m elev., from rhizosphere soil of *Lycium chinois*, 17 Oct 2018, Jing Z. Sun, living CGMCC 3.19697.

#### Teleomorph.

Undetermined.

#### Note.

Characterized by tree-like conidiophores, verticillate or in whorls of 3–4, ampulliform to lageniform phialides (3.5–7.0 × 2.6–3.4 μm), subglobose to globose conidia (2.2–2.6 × 2.0–2.4 μm). Differs from *Trichoderma lentinulae* by compact, relatively smaller phialides, and the character of pustules on SNA. Differs from *Trichoderma lixii* by shorter and wider phialides and smaller conidia.

### Trichoderma zelobreve

Taxon classificationFungiHypocrealesHypocreaceae

Jing Z. Sun & X.Z. Liu sp. nov.

6CE22E31-B776-5834-97A4-36AAE0236F30

833236

Fig. 5

#### Etymology.

Greek *zelo*, meaning emulation + *breve*, referred to *Trichoderma breve*.

#### Type.

China. Chaoyang District, Beijing, 40°0'41"N, 116°23'37"E, ca. 35 m elev., 19 Oct 2018, isolated from soil, Jing Z. Sun (HMAS 248254, holotype), ex-type culture CGMCC 3.19695.

#### Description.

**Figure 5.**
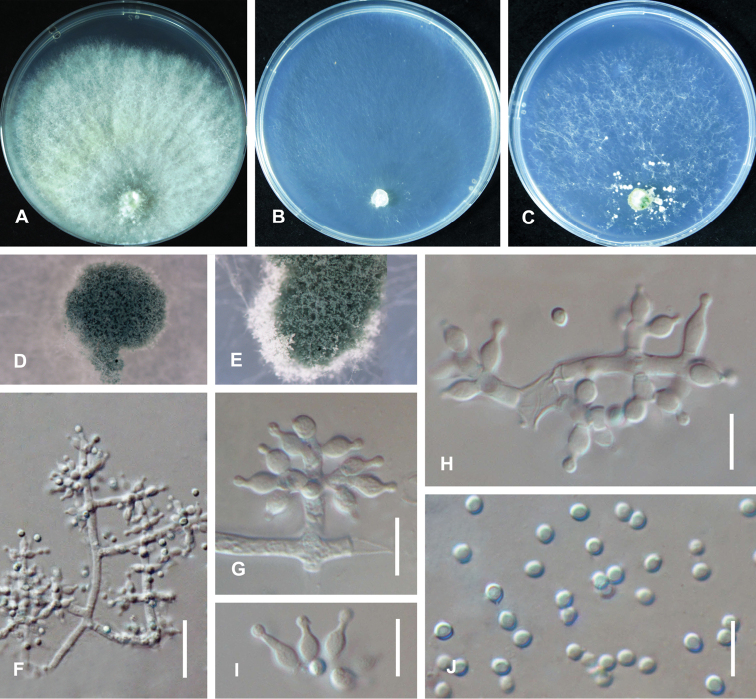
*Trichoderma zelobreve* (CGMCC 3.19695). Cultures at 25 °C after 3 days (**A** on PDA **B** on CMD**C** on SNA) **D** conidiation pustules on CMD after 10 days **E** conidiation pustules on SNA after 10 d **F** conidiophores **G, I** conidiophores and phialides **H** phialides with conidia **J** conidia. Scale bars: 25 µm (**F**); 10 µm (**G–J**).

On CMD after 72 h, colony radius covering the plate at 25 °C and 30 °C, 11–12 mm at 35 °C. Colony hyaline, indistinctly radial; Aerial inconspicuous. No diffusing pigment, not distinct odor (Fig. 5B). Conidial production noted after 5 days, starting around the original inoculum. Small pustules formed at the colony margin, first white, olivaceous after 6 d, with hairs protruding beyond the surface. Chlamydospores unobserved.

On PDA after 72 h, colony radius 55–58 mm at 25 °C, covering the plate at 30 °C, 8–9 mm at 35 °C. Colony white to yellow-white; mycelium dense and radial. Aerial conspicuous. No diffusing pigment, not distinct odor (Fig. 5A). Conidial production noted after 3 days, starting around the inoculum, effuse in the aerial hyphae, first white, turning green after 4 d. Chlamydospores unobserved.

On SNA after 72 h, colony radius 62–63 mm at 25 °C, covering the plate at 30 °C, 7–8 mm at 35 °C. Colony hyaline, regularly circular; mycelium loose. Aerial conspicuous. No diffusing pigment, not distinct odor (Fig. 5A). Conidial production noted after 2 days, starting around the inoculum, effuse in the aerial hyphae. Small pustules formed along with two concentric rings, first white, turning yellow-green after 3 d, with hairs protruding beyond the surface. Conidiophores pyramidal with opposing branches, the distance between branches relatively large (Fig. 5F). Phialides, sometimes solitary, often paired or in whorls of 2–3 (Fig. 5F); whorls typically cruciate but often nearly verticillate; rarely conidiophores nodose and phialides disposed in more or less botryose clusters (Fig. 5G, H). Phialides ampulliform to lageniform, often constricted below the tip to form a narrow neck, hyaline (Fig. 5G, H, I), (3.5–)4.0–6.0(–7.0) × (2.2–)2.6–3.2(–3.5) µm (*x̄*= 4.8× 2.9 μm, n = 30), length/width ratio (1.1–)1.4–2.1(–2.5) (*x̄*= 1.5, n = 30), base 1.4–2.1 μm (*x̄*= 1.7 μm). Conidia ovoid to subglobose, smooth, hyaline when young, becoming green to dark green with age, (2.0–)2.3–2.6(–2.9) × (1.5–)1.8–2.2(–2.5) µm (*x̄*= 2.4 × 2.0 μm, n = 30), length/width ratio (0.8–)1.1–1.4(–1.7) (*x̄*= 1.2, n = 30) (Fig. 5J). Chlamydospores unobserved.

#### Additional specimen examined.

China. Chaoyang District, Beijing, 40°0'41"N, 116°23'37"E, ca. 35 m elev., isolated from soil, 19 Oct 2018, Jing Z. Sun, living culture CGMCC 3.19696.

#### Teleomorph.

Undetermined.

**Note.** Characterized by tree-like conidiophores, branches paired or in whorls of 3–4, ampulliform to lageniform (4.0–6.0 × 2.6–3.2 μm), ovoid to subglobose conidia (2.2–2.6 × 1.8–2.2 μm). Differs from *Trichoderma breve* by shorter phialides and smaller conidia, as well as the cultural characteristics and growth rates.

## Discussion

A combination of phylogenetic, morphological, ecological, and biogeographical data has robustly resolved the taxonomy of *Trichoderma* (Jaklitsch and Voglmayr 2015; Qin and Zhuang 2016a; Sun et al. 2016; Chen and Zhuang 2017b; Qiao et al. 2018). In this study, phylogenetic analysis based on a single gene of ITS could not distinguish species of *Trichoderma* in the *Harzianum* clade from each other (Suppl. material 1: Fig. S1), which confirmed that the ITS region is not suitable for species delimitation of *Trichoderma* (Jaklitsch et al. 2012; Qin et al. 2018). Sequences of RPB2 and TEF1-α were powerful due to their suitable interspecific variations (Jaklitsch and Voglmayr 2015), and these have extensively been used in solving the taxonomy of *Trichoderma* (Jaklitsch and Voglmayr 2015; Qin and Zhuang 2016a; Chen and Zhuang 2017a, b; Qiao et al. 2018). Despite the phylogenetic analyses based on the single gene of RPB2 and TEF1-α generally revealed the phylogenetic relationship within the *Harzianum* clade (Suppl. material 1: Fig. S2, Suppl. material 3: Fig. S3), but the relationships among *T. lentinulae*, *T. xixiacum*, *T. vermifimicola*, *T. zelobreve*, and their closed taxa were not well distinct. Consideration of the universality and reliability of barcodes for species in the *Trichoderma* genus (Qiao et al. 2018), combined ITS, RPB2, and TEF1-α dataset was used for phylogenetic analysis in this study, revealing phylogenetic relationship among species in *Harzianum* clades, and suggesting that *T. lentinulae*, *T. xixiacum*, *T. vermifimicola*, and *T. zelobreve* are distinguishable from each other and species within and outside of *Harzianum* clade as well.

**Table 1.** Species, strains and their corresponding GenBank accession numbers of sequences used for phylogenetic analyses.

Species	Voucher/ culture Nos.	Origin	Substrate	GenBank accession No.		
				ITS	RPB2	TEF1-a
*Trichoderma afarasin*	CBS 130755 ^ET^	Cameroon	Soil	AY027784		AF348093
	DIS 314F	Cameroon	Wood	FJ442259	FJ442778	FJ463400
	GJS 06 98	Cameroon	Soil	FJ442630		FJ463327
*Trichoderma afroharzianum*	CBS 124620 ^ET^	Peru	*Moniliophthora roreri*	FJ442265	FJ442691	FJ463301
	CBS 466.94	Netherlands		KP009262	KP009150	KP008851
	GJS 04-193	Cameroon	Soil	FJ442233	FJ442709	FJ463298
*Trichoderma aggressivum*	CBS 100525	UK	Mushroom compost	AF057600	AF545541	AF348095
	DAOM 222156 ^ET^		Mushroom compost	AF456924	FJ442752	AF348098
	CBS 100526	Ireland	Mushroom compost	FJ442607	KP009166	KP008993
*Trichoderma alni*	CBS 120633 ^ET^	UK, England	*Alnus glutinosa*	EU518651	EU498349	EU498312
	CPK 2494			EU518652	EU498350	EU498313
	HMAS 252890				KT343763	KT343758
*Trichoderma alpinum*	HMAS 248821 ^T^	China, Sichuan	Soil	KY687906	KY687958	KY688012
	HMAS 248830			KY687912	KY687961	KY688015
	HMAS 248870			KY687953	KY687963	KY688017
*Trichoderma amazonicum*	CBS 126898 ^ET^	Peru	*Hevea brasiliensis*	HM142358	HM142367	HM142376
	IB95			HM142359	HM142368	HM142377
	LA265			HM142360	HM142369	HM142379
*Trichoderma atrobrunneum*	GJS 05-101			FJ442677	FJ442745	FJ463392
	GJS 90-254			AF443926	FJ442735	AF443943
*Trichoderma atrogelatinosum*	BMCC LU498	New Zealand				KJ871087
	CBS 237.63 ^ET^	New Zealand		MH858272	KJ842201	
	DAOM 167632					KJ871083
*Trichoderma bannaense*	HMAS 248840 ^T^	China, Yunan	Soil	KY687923	KY687979	KY688037
	HMAS 248865			KY687948	KY688003	KY688038
*Trichoderma breve*	HMAS 248844 ^T^	China, Beijing	Soil	KY687927	KY687983	KY688045
	HMAS 248845			KY687928	KY687984	KY688046
*Trichoderma brevicrassum*	HMAS 248871 ^T^		Soil	KY687954	KY688008	KY688064
	HMAS 248872		Soil	KY687955	KY688009	KY688065
*Trichoderma brunneoviride*	CBS 120928			EU518661	EU498358	EU498318
	CBS 121130 ^ET^			EU518659	EU498357	EU498316
*Trichoderma camerunense*	CBS 137272 ^ET^	Cameroon	Soil	AY027780	–	AF348107
	GJS 99 231			AY027783		AF348108
*Trichoderma catoptron*	DAOM 232830				KJ842166	KJ871245
	GJS 02 76 ^ET^	Sri Lanka	Wood	AY737766		AY737726
*Trichoderma ceramicum*	CBS 114576			FJ860743	FJ860531	FJ860628
*Trichoderma cerinum*	BMCC LU784					KJ871244
	DAOM 230012 ^ET^	Nepal		KC171336	KJ842184	KJ871242
*Trichoderma christiani*	CBS 132572 ^ET^	Spain			KJ665244	KJ665439
	S93				KJ665245	KJ665442
*Trichoderma cinnamomeum*	GJS 96-128				AY391916	AY391977
	GJS 97-233				AY391919	AY391978
*Trichoderma cinnamomeum*	GJS 97-237 ^ET^	USA, Missour	Decaying wood	AY737759	AY391920	AY737732
*Trichoderma compactum*	CBS 121218			AY941822	KF134789	KF134798
*Trichoderma concentricum*	HMAS 248833 ^T^	China, Hubei	Soil	KY687915	KY687971	KY688027
	HMAS 248858			KY687941	KY687997	KY688028
*Trichoderma corneum*	GJS 97-82 ^ET^	Thailand			KJ665252	KJ665455
*Trichoderma endophyticum*	CBS 130729 ^ET^	Ecuador	*Theobroma gileri*	FJ442243		FJ463319
	GS 2014a			FJ884177		FJ967822
*Trichoderma epimyces*	CBS 120534 ^ET^	Austria		EU518663	EU498360	EU498320
	CPK 1980			EU518662	EU498359	EU498319
	CPK 2487 ^ET^			EU518665	EU498361	EU498322
*Trichoderma estonicum*	GJS 96-129			AY737767	AF545514	AF534604
*Trichoderma guizhouense*	DAOM 231435			EF191296		EF191321
	HGUP0038 ^T^			JN191311	JQ901400	JN215484
	S628				KJ665273	KJ665511
*Trichoderma harzianum*	CBS 226.95 ^ET^	U.K.	Soil	AJ222720	AF545549	AF348101
	CBS 227.95			AF057605		AF348100
	GJS 05 107			FJ442679	FJ442708	FJ463329
	IMI 359823			EF113587		AF348092
*Trichoderma hausknechtii*	CBS 133493	France			KJ665276	KJ665515
						
					
*Trichoderma helicolixii*	CBS 133499 ^ET^	Spain			KJ665278	KJ665517
*Trichoderma helicolixii*	CBS 135583				KJ665277	KJ665516
*Trichoderma hengshanicum*	HMAS 248852 ^T^	China, Hubei	Soil	KY687935	KY687991	KY688054
	HMAS 248853			KY687936	KY687992	KY688055
*Trichoderma hirsutum*	HMAS 248834 ^T^	China, Hubei	Soil	KY687916	KY687972	KY688029
	HMAS 248859			KY687942	KY687998	KY688030
*Trichoderma hunanense*	HMAS 248841 ^T^	China, Hunan	Soil	NR_154571	KY687980	KY688039
	HMAS 248867			KY687950	KY688005	KY688040
*Trichoderma ingratum*	HMAS 248822 ^T^	China, Sichuan	Soil	KY687917	KY687973	KY688018
	HMAS 248827			KY687909	KY687966	KY688021
	HMAS 248873			KY687956	KY688010	KY688022
*Trichoderma inhamatum*	CBS 273.78 ^ET^	Colombia	Soil	FJ442680	FJ442725	AF348099
*Trichoderma italicum*	CBS 132567				KJ665282	KJ665525
	S15 ^ET^	Italy			KJ665283	KJ665526
*Trichoderma lentiforme*	CBS 100542 ^ET^	French Guiana	Decorticated wood	AF469189	–	AF469195
	DIS 253B			FJ442619	FJ442756	FJ851875
	DIS 94D			FJ442615	FJ442749	FJ463379
***Trichoderma lentinulae***	HMAS 248256 ^T^	**China**	*** Lentinula ***	**MN594469**	**MN605867**	**MN605878**
	**CGMCC 3.19848**	**China**	*** Lentinula ***	**MN594470**	**MN605868**	**MN605879**
	**CGMCC 3.19849**	**China**	*** Lentinula ***	**MN594471**	**MN605869**	**MN605880**
	**CGMCC 3.19699**	**China**	**Soil**	**MN594478**	**MN605876**	**MN605887**
	**CGMCC 3.19670**	**China**	**Soil**	**MN594479**	**MN605877**	**MN605888**
*Trichoderma liberatum*	HMAS 248831 ^T^	China,Hubei	Soil	KY687913	KY687969	KY688025
*Trichoderma liberatum*	HMAS 248832			KY687914	KY687970	KY688026
*Trichoderma linzhiense*	HMAS 248846 ^T^	China, Tibet	Soil	KY687929	KY687985	KY688047
	HMAS 248874			KY687957	KY688011	KY688048
*Trichoderma lixii*	CBS 110080 ^ET^	Thailand	Decayed *Ganoderma*	AF443920	KJ665290	AF443938
*Trichoderma neotropicale*	LA11 ^ET^			HQ022407		HQ022771
	T51			FJ884180		FJ967825
*Trichoderma parestonicum*	CBS 120636 ^ET^			FJ860803	FJ860565	
*Trichoderma parepimyces*	CBS 122768			FJ860801	FJ860563	FJ860665
	CBS 122769 ^ET^	Austria	Wood	MH863234	FJ860562	FJ860664
*Trichoderma perviride*	HMAS 273786	China,Hubei	Wood		KX026962	KX026954
*Trichoderma pinicola*	KACC 48486 ^ET^	Korea	root of Pinus densiflora	MH050354	MH025993	MH025981
	SFC20130926-S014				MH025991	MH025978
	SFC20130926-S111				MH025992	MH025980
*Trichoderma pleuroti*	CBS 124387 ^ET^	Korea	*Pleurotus* substrate	HM142363	HM142372	HM142382
	CPK 2117					EU279975
*Trichoderma pleuroticola*	CBS 124383 ^ET^	Korea	*Pleurotus* substrate	HM142362	HM142371	HM142381
	GJS 95 81			AF345948		AF348102
	TRS70 ^ET^			KP009264	KP009172	KP008951
*Trichoderma polypori*	HMAS 248855 ^T^	Hunan	Soil	KY687938	KY687994	KY688058
	HMAS 248861			KY687944	KY688000	KY688059
*Trichoderma polysporum*	S72					KJ665685
*Trichoderma priscilae*	CBS 131487 ^ET^	Spain			KJ665333	KJ665691
*Trichoderma pseudodensum*	HMAS 248828 ^T^	Hubei	Soil	KY687910	KY687967	KY688023
	HMAS 248829			KY687911	KY687968	KY688024
*Trichoderma pseudogelatinosum*	CNUN309 ^ET^	Japan	Shiitake mushroom	HM769754	HM920173	HM920202
*Trichoderma purpureum*	HMAS 273787 ^T^	China,Hubei			KX026961	KX026953
*Trichoderma pyramidale*	CBS 135574 ^ET^	Italy	*Olea europaea*		KJ665334	KJ665699
*Trichoderma rifaii*	CBS 130746	Ecuador	*Theobroma gileri*	FJ442663		FJ463324
	DIS 337F ^ET^			FJ442621	FJ442720	FJ463321
*Trichoderma rufobrunneum*	HMAS 266614 ^T^	China,Jilin	Rotten wood	KF729998	KF730010	KF729989
	isolate 8155				KF730007	KF729992
*Trichoderma rugulosum*	SFC20180301-001 ^T^			MH050353	MH025986	MH025984
	SFC20180301-002				MH025987	MH025985
*Trichoderma simmonsii*	CBS 130431	USA, Maryland	Decaying wood bark	AF443917	FJ442757	AF443935
	S297					KJ665711
	S7				KJ665337	KJ665719
*Trichoderma simplex*	HMAS 248842 ^T^	China, Guangxi	Soil	KY687925	KY687981	KY688041
	HMAS 248860			KY687943	KY687999	KY688042
*Trichoderma solum*	HMAS 248847			KY687930	KY687986	KY688049
	HMAS 248848 ^T^	China, Hubei	Soil	KY687931	KY687987	KY688050
	HMAS 248849			KY687932	KY687988	KY688051
*Trichoderma stramineum*	CBS 114248 ^ET^	Sri Lanka	Decaying wood	AY737765	AY391945	AY737746
	TAMA 0425			AB856609	AB856748	AB856675
*Trichoderma tawa*	CBS 114233 ^ET^	Thailand	Decaying bark	AY737756	AY391956	FJ463313
	DAOM 232841				KJ842187	EU279972
*Trichoderma tenue*	HMAS 273785 ^ET^	China,Hubei	Wood		KX026960	KX026952
*Trichoderma tomentosum*	DAOM 171918			AY605715		AY605759
	DAOM 178713a ^ET^	Canada, Ontario	*Ulmus* wood	EU330958	AF545557	AY750882
	DAOM 234236			EU280083		EU279971
*Trichoderma velutinum*	DAOM 230013 ^ET^	Nepal	Soil	AF149873	JN133569	AY937415
	HMAS 273865 ^T^	China, Heilongjiang	Soil		KX026965	KX026957
***Trichoderma vermifimicola***	**CGMCC 3.19850**	**China**	**Compost**	**MN594472**	**MN605870**	**MN605881**
	**HMAS 248255** ^T^	**China**	**Compost**	**MN594473**	**MN605871**	**MN605882**
***Trichoderma xixiacum***	**HMAS 248253** ^T^	**China**	**Soil**	**MN594476**	**MN605874**	**MN605885**
	**CGMCC 3.19698**	**China**	**Soil**	**MN594477**	**MN605875**	**MN605886**
*Trichoderma zayuense*	HMAS 248835 ^T^	China,Tibet	Soil	KY687918	KY687974	KY688031
	HMAS 248836			KY687919	KY687975	KY688032
***Trichoderma zelobreve***	**HMAS 248254** ^T^	**China**	**Mushroom**	**MN594474**	**MN605872**	**MN605883**
	**CGMCC 3.19696**	**China**	**Soil**	**MN594475**	**MN605873**	**MN605884**
*Trichoderma zeloharzianum*	YMF 1.00268 ^ET^	China,Yunan	Soil	MH113932	MH158996	MH183181

*Trichoderma lentinulae* was phylogenetically close to *T. xixiacum* and *T. lixii* but represents a taxon (Fig. 1). Morphologically, it differed from *T. xixiacum* in producing less frequently lageniform phialides with inequilateral to a strongly-curved apex. The conidia of *T. lentinulae* are usually more slender (*x̄*= 2.0), than those of *T. xixiacum* (*x̄*= 1.8). In addition, the conidia of *T. lentinulae* (length/width ratio, *x*‒= 1.2) are slightly more slender than *T. xixiacum* (length/width ratio, *x*‒= 1.1). The two species also differ from each other in their cultural characteristics and growth rates (Figs 2A–C, 4 A–C). *Trichoderma lentinulae* differed from *T. lixii* in producing less frequently lageniform phialide with inequilateral to a strongly-curved apex. Additionally, *T. lentinulae* forms 2–5 apex phialides on the main axis (Fig. 2F, I) in contrast to 2–4 apex phialides of *T. lixii* (Chaverri et al. 2015). *Trichoderma lentinulae* is also clearly distinguished from *T. lixii* (phialides, 6.5–3.5 μm; conidia, 3.0–2.7 μm)(Chaverri et al. 2015) in producing shorter phialides (*x̄*= 4.5 × 3.0 μm) and smaller conidia (*x̄*= 2.5 × 2.2 μm). *Trichoderma vermifimicola* was phylogenetically associated with *T. simmonsii* (Fig. 1). Morphologically, it is hard to distinguish *T. vermifimicola* from *T. simmonsii* , because both form similar tree-like conidiophores, ampulliform to lageniform phialides and ovoid to subglobose conidia, but phialide whorls of *T. vermifimicola* were often nearly verticillate rather than cruciate in *T. simmonsii* (Chaverri et al. 2015). Furthermore, *T. simmonsii* grew fast (PDA 25–55 mm, SNA 10–35 mm) at 35 °C than *T. vermifimicola*. Additionally, the length/width ratio phialide of *T. vermifimicola* is larger (*x̄*= 2.4) than that of *T. simmonsii* (*x̄*= 1.9) (Chaverri et al. 2015), and *T. vermifimicola* also produces smaller conidia (*x̄*= 2.4 × 2.2 μm) (Fig. 3) than *T. simmonsii* (3.0–2.7 μm) (Chaverri et al. 2015). *Trichoderma zelobreve* was closely related to *Trichoderma breve* in the multi-gene phylogenetic analysis (Fig. 1). Morphologically, both fungi have short phialides, however, *T. zelobreve* differs from *T. breve* by producing shorter and narrower phialides (4.0–6.0 × 2.6–3.2 μm) than that of *T. breve* (6.7–10.0 × 2.8–3.9 μm) (Chen and Zhuang 2017a). The conidia of *T. zelobreve* are smaller (*x̄*= 2.4 × 2.0 μm) than those of *T. breve* (*x̄*= 3.0 × 2.8 μm). Additionally, *T. zelobreve* does not form a zonate colony on CMD, PDA, and SNA, whereas the colony of *T. breve* presents concentric zones on CMD and PDA and finely concentric zones on SNA (Chen and Zhuang 2017a). In a previous study, the phylogenetic analysis indicated that *T. breve* was a sister taxon of *T. bannaense*, but morphologically more similar to *T. harzianum* (Chen and Zhuang (2017a). Herein, our phylogenetic analyses presented *T. breve* was associated with *T. zelobreve* (Fig. 1), resulted from the little genetic variation of sequences of ITS and TEF1-α between them. The phylogenetic analysis in Chaverri et al. (2015) presented that *T. simmonsii* was associated with *T. camerunense*. In this study, our phylogenetic analysis presented that *T. simmonsii* was phylogenetically closed to *T. vermifimicola*, and *T. camerunense* phylogenetic to *T. rifaii* (Fig. 1, Suppl. material 3: Fig. S3). In a previous study, these species were recognized as the cryptic species in under *T. harzianum* (Chaverri et al. (2015).

Currently, the *Harzianum* clade contains more than 60 species which were isolated from soil, plant tissues, and other fungi (Jaklitsch and Voglmayr 2015; Qin and Zhuang 2016a; Chen and Zhuang 2017b; Qiao et al. 2018; Sun et al. 2019a, b). Several studies have confirmed that species in this clade are important because of their mycoparasitism (Chaverri et al. 2015; Chen and Zhuang 2017a; Sun et al. 2019). When numerous biological control agents were explored deriving from species in the *Harzianum* clade (Chaverri et al. 2015, several taxa, such as *T. atrobrunneum T. pleuroti*, and *T. pleuroticola* were recognized as causing agents of “Green mold” disease of cultivated mushroom (Innocenti et al. 2019; Sun et al. 2019a, b). In this study, *T. lentinulae* was isolated from a fruiting body and the cultivated substrates of *L. edodes*, causing the decay of the host as well. How *T. lentinulae* affect the cultivation of *Lentinula edodes* is worthy of further studies. Since *T. lentinulae* was isolated from mushroom, *T. lentinulae* and *T. vermifimicola* were isolated from the mushroom spawn and substrates for earthworm cultivation, *T. xixiacum* and *T. zelobreve* were isolated from soil, confirming that species in the *Harzianum* clade have flexible nutrition modes (Chaverri and Samuels 2013; Zhang et al. 2018). The new species introduced here are not only potential candidates for biological agent exploration, but also improve our understanding of the diversity of *Trichoderma*, especially of the *Harzianum* clade in China.

## Supplementary Material

XML Treatment for Trichoderma lentinulae

XML Treatment for Trichoderma vermifimicola

XML Treatment for Trichoderma xixiacum

XML Treatment for Trichoderma zelobreve

## Supplementary materials

Supplementary material 1 Figure S1 Data type phylogenetic tree Explanation note Phylogenetic tree based on Maximum Likelihood analysis of ITS sequence dataset. *Trichoderm ceramicum*, *Trichoderma estonicum*, and *Trichoderm parastinicum* were chosen as the outgroup. Bootstrap Values higher than 70% from RAxML (BSML) (left) and Bayesian posterior probabilities greater than 0.95 (BYPP) (right) are given above the nodes. ^T^ indicates the type; ^ET^ indicates the ex-living type. Isolates obtained in this study are in red. https://binary.pensoft.net/file/460976Xin Gu, Rui Wang, Quan Sun, Bing Wu, Jing-Zu Sun

Supplementary material 2 Figure S2 Data type phylogenetic tree Explanation note Phylogenetic tree based on Maximum Likelihood analysis of RPB2 sequence dataset. *Trichoderm ceramicum*, *Trichoderma estonicum*, and *Trichoderm parastinicum* were chosen as the outgroup. Bootstrap Values higher than 70% from RAxML (BSML) (left) and Bayesian posterior probabilities greater than 0.95 (BYPP) (right) are given above the nodes. ^T^ indicates the type; ^ET^ indicates the ex-living type. Isolates obtained in this study are in red https://binary.pensoft.net/file/460978Xin Gu, Rui Wang, Quan Sun, Bing Wu, Jing-Zu Sun

Supplementary material 3 Figure S3 Data type phylogenetic tree Explanation note Phylogenetic tree based on Maximum Likelihood analysis of TEF1&alpha; sequence dataset. <em>Trichoderm ceramicum</em> was chosen as the outgroup. Bootstrap Values higher than 70% from RAxML (BSML) (left) and Bayesian posterior probabilities greater than 0.95 (BYPP) (right) are given above the nodes. <sup>T</sup> indicates the type; <sup>ET</sup> indicates the ex-living type. Isolates obtained in this study are in red. https://binary.pensoft.net/file/460979Xin Gu, Rui Wang, Quan Sun, Bing Wu, Jing-Zu Sun
